# Advances in CRISPR‐Cas9 in lineage tracing of model animals

**DOI:** 10.1002/ame2.70033

**Published:** 2025-06-10

**Authors:** Jingchao Cao, Zihang Guo, Xueling Xu, Pan Li, Yi Fang, Shoulong Deng

**Affiliations:** ^1^ College of Animal Science and Technology China Agricultural University Beijing China; ^2^ National Center of Technology Innovation for Animal Model, National Human Diseases Animal Model Resource Center, National Health Commission of China (NHC) Key Laboratory of Human Disease Comparative Medicine, Institute of Laboratory Animal Sciences, Chinese Academy of Medical Sciences and Comparative Medicine Center, Peking Union Medical College Beijing China; ^3^ College of Bee Science and Biomedicine Fujian Agriculture and Forestry University Fuzhou China; ^4^ Xianghu Laboratory Hangzhou China; ^5^ Key Lab of Animal Production, Product Quality and Security, Ministry of Education Jilin Agricultural University Changchun China

**Keywords:** cell lineage tracing, CRISPR‐Cas9, DNA barcoding, high‐throughput sequencing, xenotransplantation

## Abstract

Cell lineage tracing is a key technology for describing the developmental history of individual progenitor cells and assembling them to form a lineage development tree. However, traditional methods have limitations of poor stability and insufficient resolution. As an efficient and flexible gene editing tool, CRISPR‐Cas9 system has been widely used in biological research. Furthermore, CRISPR‐Cas9 gene editing‐based tracing methods can introduce fluorescent proteins, reporter genes, or DNA barcodes for high‐throughput sequencing, enabling precise lineage analysis, significantly improving precision and resolution, and expanding its application range. In this review, we summarize applications of CRISPR‐Cas9 system in cell lineage tracing, with special emphasis on its successful applications in traditional model animals (e.g., zebrafish and mice), large animal models (pigs), and human cells or organoids. We also discussed its potential prospects and challenges in xenotransplantation and regenerative medicine.

## Graphical abstract

Cell lineage tracing is a key technology for describing the developmental history of individual progenitor cells and assembling them to form a lineage development tree. However, traditional methods have limitations of poor stability and insufficient resolution. As an efficient and flexible gene editing tool, CRISPR‐Cas9 system has been widely used in biological research. Furthermore, CRISPR‐Cas9‐gene editing‐based tracing methods can introduce fluorescent proteins, reporter genes, or DNA barcodes for high‐throughput sequencing, enabling precise lineage analysis, significantly improving precision and resolution, and expanding its application range. For example, specific barcodes are introduced into individual cells through CRISPR‐Cas9‐mediated genome editing. Over time, as cells proliferate and divide, these barcodes accumulate spontaneous mutations in descendant cells, generating unique molecular signatures that record lineage relationships and differentiation trajectories. Single‐cell sequencing is then employed to decode the barcode sequences, enabling the reconstruction of lineage hierarchies and insights into cellular differentiation pathways.

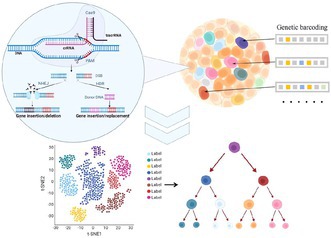

## 1 INTRODUCTION

Early mammalian embryonic development involves a complex process of cell‐fate determination that can be described by a cell lineage tree. Cell lineage is important for understanding physiological and pathological processes in multicellular organisms, including embryonic development, cell‐type differentiation, organ maintenance. and aging.^1^ Understanding early embryonic lineage establishment, the fate decision of precursor cells in germ layers and tissues, and their regulatory mechanisms not only can help to prevent early development‐related diseases and guide cell differentiation and transdifferentiation but can also provide reference for application of large animal models in human organ reconstruction and tissue regeneration.^2^ Currently, research on cell lineage tracing technology is mainly focused on traditional model animals such as nematodes,^3^ zebrafishes,^4^ and mice,^5^ with limited application in higher mammals. Large animals such as pigs, sheep, and nonhuman primates are important economic animals,^6^ with more similar characteristics to humans in many aspects, including genetics, organ structure, physiological functions, pathological responses, and metabolism. They are often ideal animal models for biomedical research, drug development, and organ reconstruction, with important research value.^7^ Therefore, expanding applications of this technique in higher mammals has great research value and broad application prospects.

In 1983, John Sulston et al. used microscopy to observe and record the division history of all cells during embryonic development of *Caenorhabditis elegans*, drawing the first complete cell lineage tree of a multicellular organism.^8^ Subsequently, radioactive elements, enzymes, and fluorescent labeling methods have been gradually applied to trace relationships of cells, but most results have been unsatisfactory. In recent years, mutation information from somatic cells has been used for lineage tracing.^9^, ^10^, ^11^ To address the low background mutation rate, scientists focused on development of DNA barcode‐based technologies, which use efficient gene editing tools such as Cre‐LoxP and CRISPR‐Cas9 to edit specific DNA barcode regions.^12^, ^13^ Cell lineage is reconstructed by recording mutations that accompany cell division.

The CRISPR‐Cas9 system has become an important tool in lineage tracing studies due to its ability to precisely introduce genetic markers.^14^ The rationale for lineage tracing using the CRISPR‐Cas9 system is to target a specific DNA sequence through recognition of a guide RNA (gRNA). Once the gRNA matches the target DNA sequence, the Cas9 protein cleaves double‐stranded DNA and generates insertion–deletion mutations (indels).^15^ Distinct indels are passed on to the offspring during cell division, forming unique genetic markers.^16^ These unique indel markers can be used for efficient cell lineage tracing. By analyzing genetic markers in each single cell by sequencing, it is possible to reconstruct the developmental path of cells, trace the entire process of cells from stem cells to specific differentiation states, and construct lineage developmental trees. This CRISPR‐Cas9‐based genetic marker approach is a powerful tool for elucidating cell development and differentiation.

## 2 RESEARCH PROGRESS AND APPLICATION OF CELL LINEAGE TRACING

Cell lineage tracing technology marks and tracks differentiation pathways of cells and their descendants, providing key evidence for understanding developmental lineage and state changes of cells, identifying differentiation pathways and interrelationships of cells in organisms, and studying tissue and organ formation. The technical core of cell lineage tracing is introduction of genetic markers and transmission of these markers to offspring during cell division, allowing reliable tracking of the development and fate of each cell along a continuous timeline. Detection of these markers, which can be physical, chemical, or genetic, allows extrapolation and mapping of the developmental trajectory of cells and determination of their differentiation pathways and eventual destinies.^17^

Early lineage tracing studies largely relied on microscopic observations of cell division. Sulston et al. produced the first complete animal cell lineage map in the 1980s by directly observing cell division in *C. elegans* embryos.^8^ Moody et al. mapped the detailed fate of each blastomere in the 32‐cell stage South African clawed frog embryo (*Xenopus laevis*).^18^ Individual blastomeres were injected with horseradish peroxidase, and all of their descendants in the late tailbud embryo (stages 32–34) were identified after histochemical processing of serial tissue sections and whole‐mount preparations. The progenies of each blastomere were characteristically distributed in both phenotype and location. Using microscopes to directly observe dynamic changes in cells in transparent or translucent organisms is intuitive and real time, allowing accurate recording of cell behaviors. However, this direct observation method is limited by the transparency and distinguishability of samples, which makes it difficult to be widely applied to complex organisms.^19^

With development of technology, dye markers have emerged. The dye is injected into target cells and is passed to the offspring during cell division, allowing lineage tracing. Honig and Hume used fluorescent carbocyanine dyes to integrate into cell membrane and diffuse throughout the entire cell, producing a stable and bright fluorescent signal, enabling them to track the lineage and migration patterns of cultured neuronal cells over time, providing insights into neuronal development and connection. Miller et al. used BrdU, a thymidine‐like compound, to label the DNA of dividing cells, which was detected by immunohistochemical techniques, allowing the identification of proliferating cells and tracking their migration and differentiation in the central nervous system (CNS).^20^ The dye labeling method is suitable for large‐scale experiments due to its simplicity and low cost. However, the dye gradually dilutes or diffuses as cells divide, resulting in unreliable labels and imprecise tracking. In addition, dye labeling may exhibit cytotoxicity, affecting normal physiological functions.^21^, ^22^

With the development of molecular biology technology, genetic marker methods have gradually replaced the traditional direct observation or dye labeling methods and have become the main tool for cell lineage tracing. Genetic marker methods use genetic engineering to track specific cells and their offspring by introducing marker genes into cells, ensuring that their expression is sustained throughout cell division.^23^, ^24^ Zhao et al. developed techniques for lineage tracing and functional studies of cellular senescence in vivo to explore the fate trajectories and specific roles of various senescent cell types during liver injury and repair. Using genetic tools based on the Dre/Cre dual recombinase system and a fluorescent reporter mouse strain, cell type‐specific p16^Ink4a+^ senescent cells were traced to reveal mechanisms of macrophages and endothelial cells in liver fibrosis; depletion of senescent macrophages alleviated liver fibrosis, whereas senescent endothelial cells contributed to tissue repair.^25^

Despite remarkable advances in the use of genetic markers for lineage tracing and the advantages of marker stability and long‐term tracking, traditional genetic markers such as fluorescent proteins, enzymes, or radioactive labeling may interfere with normal cell function and alter cell phenotype.^26^ In addition, the information capacity of these labeling methods is limited, and it is difficult to resolve the complex lineage relationships of a huge number of cells. These markers are usually one‐time static markers that fail to dynamically record changes in cells over long intervals and lack the ability to continuously generate new markers over the course of an experiment. Common genetic markers have difficulty meeting the needs of having minimal effect on cellular phenotype, containing large amounts of information, recording changes over large timescales, and consistently generating diversity in lineage tracing.^27^ Due to limitations of traditional lineage tracing technology in marker stability and tracking accuracy, CRISPR‐Cas9 system has gradually become an ideal tool for lineage tracing due to its efficient gene editing ability and diverse labeling methods.

## 3 CRISPR‐CAS9‐MEDIATED CELL LINEAGE TRACING

CRISPR‐Cas9 system provides a high‐resolution and dynamic recording technology platform for cell lineage tracing by virtue of its RNA‐guided, DNA‐targeted cutting mechanism. Single‐stranded guide RNA (sgRNA) guides Cas9 nuclease to induce double‐strand breaks (DSBs) by complementary pairing with the target DNA sequence, triggering cells to generate genetic markers through nonhomologous end joining (NHEJ) or homology‐directed repair (HDR).^28^ NHEJ‐mediated random insertion or deletion mutations (indels) form unique genetic barcodes, whereas HDR achieves precise gene insertion, thereby achieving high‐resolution tracking of cells and their progeny without interfering with the normal function of cells.^29^ These genetic markers are stably inherited during cell division. By designing multiple sgRNAs to edit multiple sites in the genome, complex and diverse genetic markers can be generated to analyze the lineage relationships of heterogeneous cell populations.^30^ In addition, the inducible CRISPR‐Cas9 system can be used to activate Cas9 at specific time points, enabling continuous recording of cell development and dynamically capturing changes in cell states over a long period of time.^5^, ^31^ Further integration of single‐cell RNA sequencing (scRNA‐seq) technology with specific genetic barcodes can generate high‐precision lineage tracing data, simplify data interpretation, and improve the accuracy and reproducibility of results.^32^, ^33^

### 3.1 CRISPR‐Cas9‐mediated cell lineage tracing techniques

In the field of cell lineage tracing research, the direct application strategy of CRISPR‐Cas9 technology is to introduce fluorescent proteins or reporter genes through gene editing to achieve visual tracking of cells and their progeny. A common method is to use the CRISPR‐Cas9 system to insert reporter genes, such as green fluorescent protein (GFP) or red fluorescent protein (RFP), at specific gene sites to activate and mark target cells and their progeny under specific conditions. Shahabipour et al. developed a DMP1 promotor‐mediated DsRed‐GFP knock‐in mesenchymal stem cell (MSC) model to integrate GFP and DsRed reporter genes into human ROSA locus using a CRISPR‐Cas9‐mediated homology‐directed knock‐in strategy, effectively tracking the differentiation process of osteoblasts.^34^ Similarly, Schmidt et al. used CRISPR/Cas9 technology to specifically edit the green fluorescent protein gene locus of *C. elegans* and successfully introduced genetic markers in >60% of cells, enabling accurate tracking of cell differentiation.^35^ This method has advantages of simplicity and the ability to label a large number of cells efficiently; however, it requires high‐design requirements for sgRNAs and reporter gene insertion sites to avoid adverse effects on host genome function.

The combination of CRISPR‐Cas9 technology and specific recombinase systems (such as the Cre‐LoxP system) can achieve more efficient lineage tracing. Schepers et al. used CRISPR‐Cas9 and Cre‐LoxP systems to construct a mouse model expressing multicolor reporter genes. This model not only achieved lineage tracing of intestinal stem cells but also revealed the role of Lgr5‐positive tumor cells in promoting the growth of intestinal adenomas.^36^ In the study of zebrafish models, researchers used CRISPR‐Cas9 technology to integrate the CreERT2 gene into the otx2 locus and achieved precise labeling and developmental pathway tracking of specific cell populations through conditional Cre enzyme induction.^37^ Although this method requires the construction of an animal model of CRISPR‐Cas9 and Cre‐LoxP systems, and the operation process is relatively complicated, it can fully record the dynamic trajectory of the developmental process and provide a powerful tool for cell lineage tracing research, demonstrating the broad application prospects of CRISPR‐Cas9 technology in in‐depth understanding and precise monitoring of cell fate and developmental processes.^38^

Second, CRISPR/Cas9 systems can generate unique genetic markers by introducing barcodes or random mutations to achieve high‐resolution cell lineage tracing. This approach involves a variety of techniques, including genome editing of synthetic target arrays for lineage tracing (GESTALT),^30^ single‐cell labeling of endogenous proteins by CRISPR‐Cas9‐mediated homology‐directed repair (SLENDR) technique,^39^ and memory by engineered mutagenesis with optical in situ readout (MEMOIR).^40^ GESTALT technology, one of the earliest approaches, uses CRISPR‐Cas9‐mediated genome editing to progressively introduce and accumulate diverse mutations in DNA barcodes over multiple rounds of cell divisions. The barcode, an array of CRISPR‐Cas9 target sites, marks cells and enables elucidation of lineage relationships via patterns of mutations shared between cells. By sampling hundreds of thousands of cells from individual zebrafish, thousands of barcoded alleles with lineage information can be generated, allowing large‐scale maps of cell lineage in multicellular systems for normal development and disease.^41^

McKenna et al. combined GESTALT with scRNA‐seq to sequence approximately 60 000 transcriptomes from the juvenile zebrafish brain and identified >100 cell types and marker genes. Using these data, they generated lineage trees with hundreds of branches that help uncover restrictions at the level of cell types, brain regions, and gene expression cascades during differentiation.^42^ Homing CRISPR Barcodes technology^31^ is a further extension of barcodes, using CRISPR‐Cas9 system to introduce “homing” barcodes at specific sites in the genome, which continuously change with cell replication and division to form unique markers. This technology relies on autologous genome, without the introduction of exogenous DNA, and has high biocompatibility and stability, making it especially suitable to study complex cell lineages and multicellular systems. This technology provides an enabling and versatile platform for in vivo barcoding and lineage tracing in a mammalian model system. In addition, modified synthetic cellular recorders integrating biological events (mSCRIBE) technology consists of a self‐targeting guide RNA (stgRNA) that repeatedly directs Cas9 nuclease activity toward the DNA that encodes stgRNA, enabling localized, continuous DNA mutagenesis and continuous evolution of target DNA sequence. This technology can record biological events during cell development and division in vivo. The core of this technology is the design and insertion of mutant sequences, with accurate recording and analysis at the single‐cell level.^43^

To further improve the diversity and labeling efficiency of barcodes, researchers have developed piggyBac (PB) transposon system combined with CRISPR‐Cas9 system for lineage tracing, which is defined as PB transposon‐mediated molecular recorder technology. The insertion of exogenous DNA barcodes into the genome through the PB transposon system enables more extensive and flexible lineage tracing. The piggyBac transposon system has advantages of high copy number and the ability to insert multiple target sequences in the genome, ensuring sufficient barcoding diversity and reducing possibility of marker overlap. Weissman et al. designed multiple piggyBac plasmids, containing three tandem gRNA sequences and corresponding target sequences. A mouse model with multiple piggyBac insertion sites was obtained by injecting transposase messenger RNA (mRNA) and piggyBac plasmids into mouse oocytes and artificial insemination with Cas9‐EGFP encoding sperm. A large number of heritable repair and tracking of multiple target sites were achieved.^27^

Eggenschwiler et al. developed piggyBac prime editing (PB‐PE) to enable sustained expression of prime editors, further demonstrating the efficiency and flexibility of PB transposon system in combination with a CRISPR system for lineage tracing. High‐resolution tracing and analysis of human cell lineages have been achieved.^44^ Chen et al. successfully tracked and transformed neocortical precursor cells by combining CRISPR‐Cas9 with PiggyBac transposase and demonstrated its potential application in neural lineage tracing.^45^ Weber et al. used PB transposon and CRISPR‐Cas9 system to trace the lineage of B‐cell lymphoma in a mouse model, demonstrating the lymphoma driver genes and further confirming the widespread application of PB transposon system in lineage tracing.^46^ In addition, Wang et al. combined CRISPR‐Cas9 system with PB transposon technology for gene editing human‐induced pluripotent stem cells (hiPSCs), achieving cell‐fate tracking of hiPSCs at the single‐cell level, highlighting advantages of the PB transposon system combined with CRISPR‐Cas9 technology.^47^

The development of cell lineage tracing technology can be divided into several key stages. In the 1980s, researchers first achieved intuitive tracking of cell dynamics using microscopes but were limited by the inefficiency of manual operations and sample throughput bottlenecks. From the 1990s to the beginning of the new century, fluorescent dyes and radioactive labeling methods significantly improved detection sensitivity but faced the inherent defect of signal diffusion and attenuation over time. With the breakthrough of genetic engineering technology, the fluorescent protein labeling and Cre‐LoxP recombination system introduced in the early 21st century pushed the tracking accuracy to the subcellular level. Subsequently, the emergence of CRISPR‐Cas9 gene editing tools completely changed the rules of the game. Its derivative technologies GESTALT and homing CRISPR achieved parallel tracking of large‐scale cell populations through multiple targeting strategies. In 2019, the research team innovatively combined CRISPR‐Cas9 with the PiggyBac transposon system to create a “molecular recording tool” that can be stably inherited, providing a long‐term solution for cross‐generational tracking studies. These progressive innovations not only deepen the understanding of the mechanism of cell‐fate determination but also provide key methodological support for analyzing developmental dynamics and tumor heterogeneity evolution. Table 1 summarizes the key lineage tracing technologies developed in different periods and their characteristics.

**TABLE 1:** Cell lineage tracing techniques.

Time	Method	Principle	Characteristics	References
1983	Direct microscopic observation	Direct observation of cell behavior in transparent or translucent organisms through a microscope and recording the cell development process	For direct observation of cell division and spatial information, suitable for clear or translucent samples and organisms with small cell numbers. Opaque organisms cannot be tracked, and observations of large organisms are not applicable and have low resolution	[8, 18]
1986	Dye labeling	Injection of fluorescent dyes or BrdU into tissues or cells to trace migration and differentiation	For tracking cells over time and identification of proliferating cells. However, the dye weakens over time, which cannot be permanently labeled and track cell fate of multiple generations of cells. It's also prone to proliferation problems	[20, 48, 49]
1999	Genetic markers	Genetic markers, such as fluorescent proteins and Cre‐LoxP systems, are introduced through genetic engineering to track cell differentiation and fate	Efficient, stable, and suitable for long‐term tracking. The operation of genetic engineering is complex, and recognition of specific cell populations is not accurate, which may interfere with gene function	[26, 50]
2013	CRISPR‐Cas9‐mediated fluorescent labeling	Fluorescent protein‐encoding genes are inserted using CRISPR‐Cas9 guide sequences to generate fluorescent tags for specific cells	Efficient labeling of multicells and tracking cell differentiation in real time. The disadvantage is that the editing efficiency is uneven, which may produce off‐target effects. The labeling efficiency varies based on different cell types and experimental conditions	[34, 35, 51]
2014	CRISPR‐Cas9 combined with Cre‐LoxP system	The Cre‐LoxP system is integrated into the genome for efficient cell lineage tracing in combination with CRISPR/Cas9	This method can efficiently demonstrate cell differentiation pathways and fate and is suitable for tracing complex organisms. The disadvantage is that Cre‐LoxP system has limited marker locations, which cannot achieve whole‐genome labeling. The integration efficiency is also not high	[36, 37, 52]
2016	GESTALT	Using CRISPR‐Cas9 system to insert barcode sequences at target gene locations, accumulation of gene editing events during cell development, such as insertion or deletion mutations, and recording cell differentiation information	Cell lineage maps are constructed from cumulative gene editing events, but the limited number of barcodes makes it difficult to fully parse all the information in a complex sample	[30, 38]
2017	Homing CRISPR barcodes	The stgRNA guides self‐localization and causes mutations, which can record the division history during cell development. This method realizes cell lineage tracking and reconstruction	Cell development changes can be recorded without exogenous DNA insertion, which is suitable for diversity research. However, marker efficiency is limited, genome integration is unstable, and comprehensive tracking is elusive	[31]
2017	SLENDR	The CRISPR‐Cas9 system and homologous recombination template are introduced into neural progenitors through electroporation to achieve precise localization and tracking of specific proteins in neural tissue	Provides protein localization and cell differentiation tracking with high resolution, which is suitable for mammalian brain research. The operation is demanding, and the efficiency of electroporation is affected by many factors. It is mainly suitable for actively mitotic cell types, with limited application in mature neurons	[39]
2017	MEMOIR	The introduction of editable barcodes inside cells via CRISPR‐Cas9 system combined with smFISH technology to read genetic information directly inside cells	Single‐cell level event recording and analysis are achieved, which are suitable for tracking the differentiation of individual cells. However, it is difficult to scale up applications in large samples or entire organs	[40, 53]
2019	PiggyBac transposon system combined with CRISPR‐Cas9	PiggyBac transposon is used to introduce target genes or markers into the cell genome for multitarget site tracking	Can track complex cell lineage and record changes in multitarget sites, which is suitable for studying the development process of multicell types. However, efficiency of transposon integration is limited and may cause insertion mutations and affect function	[44, 46]

Abbreviations: GESTALT, genome editing of synthetic target arrays for lineage tracing; MEMOIR, memory by engineered mutagenesis with optical in situ readout; SLENDR, single‐cell labeling of endogenous proteins by CRISPR‐Cas9‐mediated homology‐directed repair.

Table 1 summarizes the above cell lineage tracing methods, including their names, principles, and characteristics of these methods.

### 3.2 CRISPR‐Cas9‐mediated cell lineage tracing information reading technique

Whether using CRISPR‐Cas9 system to generate barcodes or combining CRISPR‐Cas9 with exogenous barcodes for lineage tracing, barcode information must be read and aligned to construct the lineage tree. Barcode reading has undergone many changes, with CRISPR‐Cas9‐mediated lineage tracing information reading technology from polymerase chain reaction (PCR) amplification and high‐throughput sequencing (NGS) to scRNA‐seq facilitating lineage tracing.

Early studies mainly relied on PCR amplification technology to detect barcodes generated by CRISPR‐Cas9. Yang et al. developed a “KP‐Tracer” mouse model with an engineered lineage tracing system that allowed induction of Kras and Trp53 oncogenic mutations using the CRISPR‐Cas9 system in individual lung epithelial cells. By optimizing PCR amplification technology, continuous and comprehensive monitoring of the process through which a single cell harboring an oncogenic mutation evolves into an aggressive tumor revealed the evolutionary path and clonal diversity of tumors, providing insights into cellular behavior of tumors during evolution.^54^

Similarly, Li et al. elucidated contributions of nonmyocytes in cardiovascular regeneration by identifying and tracing the differentiation trajectory of nonmyocyte populations during cardiac regeneration through PCR amplification of CRISPR‐Cas9‐induced barcodes.^55^ Although PCR amplification methods achieved successful cell lineage tracing, their inefficiency and insufficient throughput limit application in complex multicellular systems, prompting a search for more efficient technologies.

To improve throughput and efficiency, researchers have begun to adopt high‐throughput sequencing (NGS) technology. Liu et al. used NGS to sequence CRISPR‐Cas9 barcodes introduced during zebrafish embryonic development and successfully constructed a complete cell developmental lineage model, revealing cell fate during zebrafish development.^4^ Ye et al. used CRISPR‐Cas9 system to introduce endogenous barcodes into the zebrafish genome and constructed a zebrafish cell lineage development model by NGS, demonstrating the powerful application potential of NGS technology in multicellular systems.^56^ In addition, NGS technology can effectively capture changes in gene expression and differentiation of cells during therapy, particularly in the CAR‐T cell therapy and stem cell therapy, helping to identify key cell populations related to therapeutic efficacy and revealing cellular heterogeneity and dynamics during therapy.^57^

**FIGURE 1:**
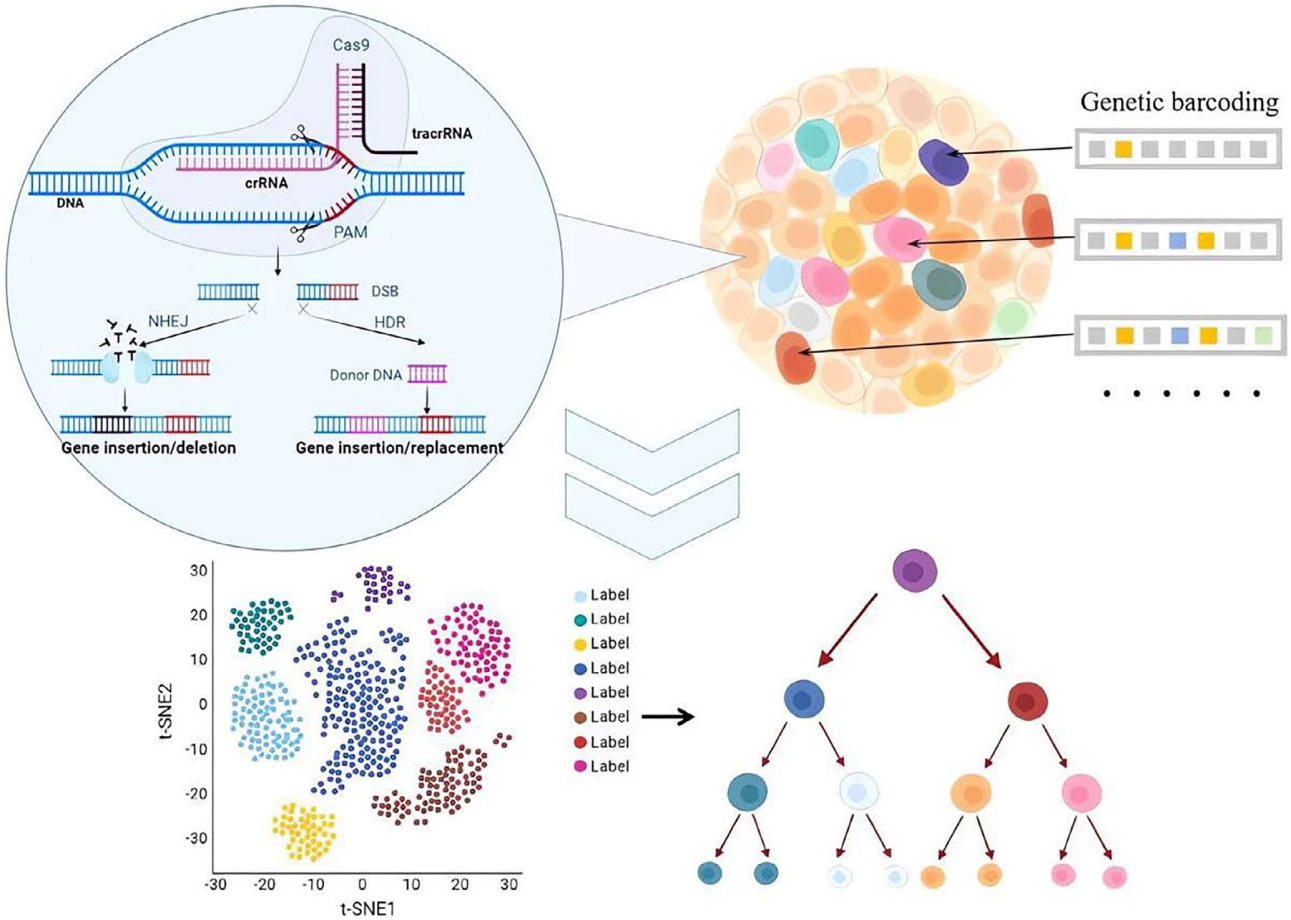
Lineage tracing via CRISPR‐Cas9 coupled with single‐cell sequencing. This figure illustrates the process of cell lineage tracing using CRISPR‐Cas9 technology in combination with single‐cell sequencing. Specific barcodes are introduced into individual cells through CRISPR‐Cas9‐mediated genome editing. Over time, as cells proliferate and divide, these barcodes accumulate spontaneous mutations in descendant cells, generating unique molecular signatures that record lineage relationships and differentiation trajectories. In the illustration, different colors represent cell populations with distinct barcodes, whereas different shapes indicate various cell types. Single‐cell sequencing is then employed to decode the barcode sequences, enabling the reconstruction of lineage hierarchies and insights into cellular differentiation pathways.

Although NGS technology has greatly improved the efficiency of cell lineage tracing, it cannot simultaneously obtain transcriptional status of cells. However, scRNA‐seq is a breakthrough in cell lineage tracing (see Figure 1). scRNA‐seq technology has been applied to isolated cells to simultaneously read out cell barcodes and transcriptomes, contributing to a better understanding of distinct fates and differentiation of histologically identical neighboring cells during development.^58^ The combination of barcodes and scRNA‐seq can reveal the gene activity and state of individual cells in time and space,^59^, ^60^ ultimately unraveling the process by which a single cell develops into the complex tissues and organs of an animal. Pellecchia et al. used scRNA‐seq to construct a lineage map of resistance to anti‐EGFR therapy in triple‐negative breast cancer, which successfully revealed the dynamic changes in tumor clones and mechanisms of tumor resistance.^61^ Xie et al. combined scRNA‐seq with the CRISPR‐Cas9 system to construct a detailed spatial and temporal lineage map at the single‐cell level in mouse brain development, providing a new perspective of nervous system development.^62^ Hwang et al. successfully constructed a high‐resolution lineage map of mammalian cell development by combining CRISPR‐Cas9 barcodes with scRNA‐seq technology, demonstrating the potential of scRNA‐seq for high‐precision lineage tracking.^63^

### 3.3 Limitations and optimization of CRISPR‐Cas9‐mediated cell lineage tracing technique

CRISPR‐Cas9 system has revolutionized cell lineage tracing by enabling precise genetic editing. However, there are several limitations.

Off‐target effects of CRISPR‐Cas9 system can introduce unintended mutations at nontargeting sites and produce false lineage signals or confounding results to distort lineage trees. High‐fidelity Cas9 variants^64^ (e.g., eSpCas9 or HiFi Cas9) and optimized sgRNA^65^, ^66^, ^67^ can minimize off‐target effects and improve reproducibility.

CRISPR‐Cas9 editing can induce cellular stress, DNA damage, or apoptosis, particularly in sensitive cell types. This may alter normal cell behavior or reduce the pool of traceable cells, skewing lineage data. For example, genome editing by CRISPR‐Cas9 induces a p53‐mediated DNA damage response and cell cycle arrest in immortalized human retinal pigment epithelial cells, suggesting that p53 function should be monitored when developing the CRISPR‐Cas9 technique.^68^ Several newly developed precise CRISPR genome editing techniques without double‐strand breaks, such as prime editing, may reduce off‐target effects and cytotoxicity.^69^

The efficiency of CRISPR‐Cas9 editing varies across cell types and tissues. Inconsistent editing can result in missed critical cell relationships and incomplete lineage tracing.^30^ In highly dynamic tissues, CRISPR‐Cas9‐mediated lineage tracing typically provides a snapshot rather than real‐time tracking of cell relationships. This makes it difficult to capture transient or rapidly changing cell states, which can lead to gaps in understanding the developmental or regenerative processes. Chan et al. developed a flexible, high‐information, multichannel molecular recorder with a single‐cell readout by combining lineage information with scRNA‐seq profiles. This approach enables massively parallel, high‐resolution recording of lineage and other information in mammalian systems.^70^

Designing and implementing CRISPR‐Cas9‐mediated lineage tracing experiments require sophisticated tools, such as single‐cell sequencing and computational analysis. The technical complexity can limit accessibility and scalability. In addition, the large‐scale data generated from barcode sequencing and analysis require advanced computational tools and expertise. Errors in data analysis can lead to incorrect lineage reconstructions.^42^ Multidimensional lineage tracing combining CRISPR‐Cas9 with other omics technologies (e.g., transcriptomics, proteomics) can be technically challenging. Advanced computational tools for data analysis and lineage reconstruction can integrate real‐time imaging and single‐cell omics technologies.^71^

Although CRISPR‐Cas9 has significantly advanced lineage tracing, overcoming these limitations will be crucial for its broader and more accurate application in developmental biology, cancer research, and regenerative medicine.

## 4 CELL LINEAGE TRACING TECHNOLOGY

**FIGURE 2:**
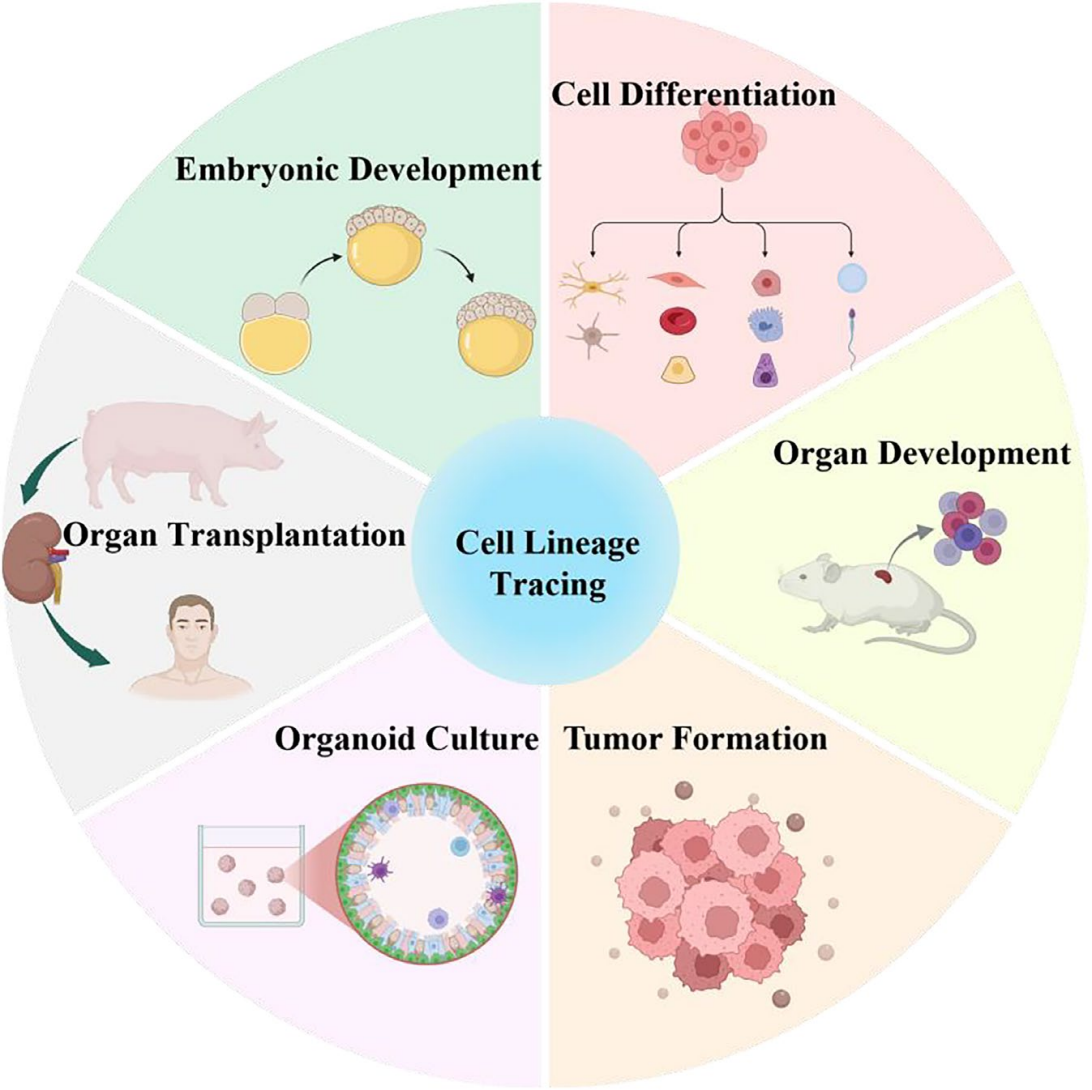
The application of cell lineage tracing technology. This figure illustrates the main applications of cell lineage tracing technology in various biological research fields. These applications include embryonic development, where lineage tracing helps track the origin and differentiation of cells in early developmental stages: Cell differentiation, allowing the study of cell fate decisions and lineages; organ development, providing insights into the formation, structural organization, and cellular interactions within organs; tumor formation, promoting the understanding of cancer cell proliferation, progression, and heterogeneity; organoid culture, enabling the study of tissue‐specific lineage dynamics in vitro; and organ transplantation, assisting in tracking the integration, function, and fate of transplanted cells or tissues. Each section represents a unique field where lineage tracing plays a vital role in advancing biological and medical knowledge.

Cell lineage tracing technology is used to study cell division dynamics, cell‐fate decisions, and spatial organization, facilitating tracking cell differentiation and state change during development, homeostasis, or disease. It facilitates understanding differentiation of specific cells into tissues and organs during early development, revealing mechanisms of cell‐fate decisions. The following are some applications of cell lineage tracing technique and a brief introduction to existing models (see Figure 2).

### 4.1 Application of cell lineage tracing technology in cultured cells

To determine whether genome editing of synthetic target arrays for lineage tracing (GESTALT) could be used to reconstruct lineage relationships, McKenna et al. applied it to a designed lineage in cell culture.^30^ EGFP‐positive HEK293 cells were transfected with the CRISPR‐Cas9 system to induce two rounds of mutations in barcodes. Clones derived from single cells were expanded, sampled, and split. Unique molecular identifiers (UMIs; 10 bp) were incorporated during amplification of barcodes for each clonal population. Each UMI tags the single barcode present within each cell, thereby allowing for correction of subsequent PCR amplification bias and enabling each UMI‐barcode combination to be interpreted as derived from a single cell. Subsequently, the maximum parsimony approach was used to reconstruct the lineage relationships among all alleles observed in the experiment, resulting in a lineage tree. Each clonal population was cleanly separated upon lineage reconstruction, with >99.7% of cells accurately placed into major clade of each lineage. These results demonstrated that GESTALT can be used to capture and reconstruct cell lineage relationships in cultured cells.

### 4.2 The application of cell lineage tracing technology in model animals

Early lineage tracing studies were conducted in *C. elegans* ^12^ and *X. laevis* ^13^ using direct microscopic observation. With the development of labeling methods, scientists have precisely marked cells in zebrafish embryos, adults, and brains using Cre‐LoxP system, CRISPR‐Cas9 system, and NGS technology, tracing development of specific cell populations.^3^, ^31^, ^36^, ^51^

Liu et al. developed a lineage tracing system based on single‐base substitution mutation, referred to as substitution mutation‐aided lineage tracing system (SMALT). This system has a large space of markers, with >1000 potential mutation sites. Each individual barcode can record >20 mutations on average, making it suitable for lineage tracing in complex multicellular organisms. The SMALT system used deaminase (AID) to target DNA barcode sequences through the functional domain of iSceI proteins. Cytosine (C) is changed to a uracil (U) through deamination. Upon DNA replication, the uracil pairs with the adenine (A), resulting in C‐to‐T mutations. By constructing SMALT transgenic *Drosophila* strains, researchers have accomplished the first reliable description of cell lineage relationships during embryonic development of complex multicellular organisms at single‐cell levels. This method can infer the population history in the development process of each organ from cell lineage tree. Different organs exhibit unique population history characteristics that are highly consistent across individuals. Based on these results, the cell lineage tree contains the underlying logic and provides new technical approaches and research methods for reconstructing the process of embryonic development in complex multicellular organisms. When combined with single‐cell multiomics methods, it is expected to conduct quantitative analysis of the core problems in developmental biology such as mammalian organ development and cell fate switch.^2^

### 4.3 Application of cell lineage tracing technology in diseases

Cell lineage tracing technology is a powerful tool used to understand human diseases, particularly in the fields of cancer, regenerative medicine, and developmental disorders.

A tumor can usually be treated by surgery or other methods when it is confined to a part of the body. Most cancer‐related deaths are due to the propensity of metastasis. To monitor metastasis of tumor cells in real time, Weissman et al. drew a detailed tumor cell lineage tree in a lung cancer mouse xenograft model using CRISPR‐Cas9 system. By studying branches of this lineage tree, individual tumor cells can be traced to determine the time point at which they become abnormal and spread their progeny to other parts of body. The combination of single‐cell lineage tracing and RNA‐seq can capture diverse metastatic behaviors of mouse lung cancer xenografts and the driving factors, helping to unravel mechanisms of tumor spread and progression.^72^ Nicholas et al. reported “DAISY‐barcode,” a high‐resolution barcodes design tool based on CRISPR system generated by online deep machine learning and optimization. This tool realizes the integration of gene expression information and cell lineage information at the single‐cell level.

Using another CRISPR nuclease Cas12a, in addition to Cas9, to edit DAISY‐barcode enables tracking a single cell and thousands of its descendants. To demonstrate this new single‐cell barcoding tool in disease‐related fields such as cancer, researchers tracked the evolution of >47 000 human melanoma cells. Through the integrated analysis of single‐cell state and lineage, they determined that EZH2 may regulate gene expression involved in the cell‐state transitions of melanoma cells into dedifferentiated cell states composed of high‐memory genes. This finding revealed that the PRC2 complex, which consists of EZH2 and SUZ12, may be the key epigenetic mechanism affecting the evolutionary path of melanoma cells. Based on these results, EZH2 and the PRC2 complex could serve as potential therapeutic targets for melanoma and other related tumors. This single‐cell barcoding technology can be applied to more complex animal models in vivo to explore new mechanisms driving cancer immune evasion and metastasis.^73^

Understanding the early mechanisms of tumorigenesis is very important for precision early screening, intervention, and prevention of cancer. In the 1970s, Peter Nowell proposed the classic clonal evolution theory, which holds that tumors are initiated by a single cell with an oncogene mutation, and eventually form malignant tumors after continuous clonal expansion and selection. This theory emphasizes that tumors have a single origin but develop a high degree of genetic diversity.^74^

However, understanding of the precancerous process of tumors at the earlier stages is very limited. For example, is there “polyclonal origin” in which multiple independently originating, simultaneously expanding cell populations that collectively drive tumor development? The hypothesis of monoclonal or polyclonal origin in early tumor evolution has been debated. On the one hand, genome sequencing appears to support Nowell's idea of monoclonal origin. Sequencing data, on the contrary, only show that the vast majority of cancer cells belong to the same clone. Under cell competition, other clones are difficult to detect after the tumor has begun to grow. The field cancerization theory also puts forward the opposite view that microenvironment generating cell clones should be able to generate more than one cell clone. In 2022, the study by Bingjie Chen et al. also supported the idea of polyclonal origin.^75^

Lu et al. developed the first base editor‐based lineage tracing technology in mammals, called SMALT, which combines cytidine deaminase (AID), artificial DNA barcoding sequences, and a Tet‐On inducible expression system to enable high‐precision lineage tracing at large‐scale single‐cell resolution in mice. Genomic sequencing data from two mouse models of colorectal cancer and a cohort of human patients with colorectal cancer revealed that most tumors had polyclonal origin with a low mutational burden and copy number variation. The pathological features also revealed that monoclonal tumors had more genomic variation, larger size, and higher malignancy than polyclonal tumors, suggesting that monoclonal tumors may represent a more “advanced” stage of tumorigenesis.^76^

Based on these findings, they proposed a new model of early tumor evolution called “polyclonal to monoclonal transition” in tumorigenesis, which suggested that early tumor lesions often have multiple independent clonal origins that jointly promote tumor progression in the early stage of tumorigenesis. As the tumor progresses, these clones are gradually replaced by a dominant clone, transforming into a monoclonal tumor. This model highlights the importance of specific genetic variants and microenvironment changes, as well as cell‐to‐cell interactions, and proposes new strategies for tumor prevention by targeting cell–cell communication for early intervention. This study provides a new idea for early tumor screening, risk prediction and individualized targeted early intervention, deepens the understanding for the origin and evolution of tumor heterogeneity, and promotes the development of cancer precision medicine.

Cell lineage tracing helps map the differentiation pathways of stem cells, which can track how stem cells contribute to tissue repair after injury, aiding in the development of stem cell‐based regenerative therapies. Lineage tracing for a wide range of tissue stem/progenitor cells revealed that these stem cells are lineage restricted and contribute to restore the mouse distal digit, mimicking digit growth during development. These results demonstrate that tissue stem cells are an evolutionarily conserved cellular mode for limb regeneration after amputation. Intestinal crypts display robust regeneration upon injury.^77^ By single‐cell lineage tracing of dedifferentiating enterocytes, Tetteh et al. conclude that the highly proliferative, short‐lived enterocyte precursors serve as a large reservoir of potential stem cells during crypt regeneration.^78^

Lineage tracing can track the development and degeneration of neurons, helping to understand diseases like Alzheimer's, Parkinson's, and amyotrophic lateral sclerosis (ALS), and can also reveal the role of glial cells in neurodegenerative diseases and brain injury repair. Using single‐cell lineage tracing, Keren‐Shaul et al. comprehensively map all immune populations in wild‐type and AD‐transgenic (Tg‐AD) mouse brains and describe a novel microglia type associated with neurodegenerative diseases (disease‐associated microglia [DAM]). This unique microglia type has the potential to restrict neurodegeneration, which may have important implications for future treatment of AD and other neurodegenerative diseases.^79^

Lineage tracing can monitor the immune cells during infections, providing insights into the responses of the immune system to pathogens like viruses or bacteria. During an immune response, CD8+ T cells are recruited to provide protection. Gerlach et al. used in vivo fate mapping of mouse T cells with a defined specificity during a bacterial infection to show that the dynamics of the single‐cell response are not uniform.^80^ Schlitzer et al. analyzed the differentiation and functional heterogeneity of macrophages and dendritic cells in bacterial infections, such as listeria infections, using scRNA‐seq and lineage tracing. Macrophages were found to differentiate into subpopulations that are involved in pathogen clearance and tissue repair after infection.^81^ Schultheiß et al. created a repository of >14 million B‐ and T‐cell receptor (BCR and TCR) sequences from the blood of COVID‐19 patients and analyzed adaptive immunity in patients with active infection or after recovery. SARS‐CoV‐2‐specific T‐cell responses were driven by TCR clusters shared between patients with a characteristic trajectory of clonotypes and traceability over the disease course. These data provide fundamental insight into adaptive immunity to SARS‐CoV‐2 and serve as a valuable resource to inform urgently needed therapeutic concepts and vaccine development.^82^

Lineage tracing can identify the accumulation of senescent cells, which contribute to aging and age‐related diseases like osteoporosis or cardiovascular disease. Using lineage tracing, Farr et al. have tagged senescent cells in bone (osteoblasts and osteoclasts) and traced their role in osteoporosis. The results showed that the accumulation of senescent cells in bone contributes to the development of osteoporosis by decreasing bone formation and increasing bone resorption.^83^ In cardiovascular disease, Childs et al. used lineage tracking to mark senescent cells in vascular endothelial cells and smooth muscle cells. They find that foamy macrophages with senescence markers accumulate in the subendothelial space at the onset of atherosclerosis and drive pathology by increasing expression of key atherogenic and inflammatory cytokines and chemokines. In advanced lesions, senescent cells promote features of plaque instability, including elastic fiber degradation and fibrous cap thinning, by heightening metalloprotease production. These results demonstrate that senescent cells are key drivers of atheroma formation and maturation.^84^

In summary, cell lineage tracing technology is a transformative tool for understanding the cellular and molecular mechanisms underlying human diseases, offering new avenues for diagnosis, treatment, and prevention.

### 4.4 Application of cell lineage tracing technology in a mouse model

Mouse models have an important role in biomedical research. The high similarity in genome between the mouse and human (~ 85%), combined with the advantages of rapid reproduction, low cost of rearing, and easy genetic manipulation, makes the mouse an ideal model for studying mechanisms and treatment strategies of human disease.^85^ In recent years, with the rapid development of gene editing technology, the application of mouse models has been further expanded, providing a valuable tool for in‐depth understanding of the molecular basis of diseases. Mouse models have had a key role in the application of lineage tracing technologies, particularly in major advances of gene editing and cell tracking.^86^ Using mouse models, we are able to study the fate and evolution of cells during development, regeneration, and disease in detail. Several mouse lineage tracing models based on CRISPR‐Cas9 technology and their applications are described in detail below, demonstrating their potential.

The multicolor mouse model (Confetti model) is a tool that uses Cre‐loxP system to randomly activate various fluorescent protein‐encoding genes in specific tissues or cells, labeling cells multiple colors and helping to distinguish between cell lineages and track their progeny.^87^ Snippert et al. used various color markers to trace the progeny of individual stem cells to understand their contributions to tissue repair when studying intestinal epithelial regeneration.^88^ Lotte Bruens et al. used a Confetti mouse model to provide visual evidence of crypt fission and fusion in the adult mouse gut, demonstrating the dynamic behavior of intestinal crypts that contribute to tissue homeostasis and regeneration.^89^ In addition, to further explore the applicability of this model in other tissues or organs, the Confetti mouse model has also been widely used to study stem cell behavior and tumors in mammary gland and skin.^90^, ^91^ These studies help to reveal the complex mechanisms of tissue regeneration and cell differentiation.

In addition to the multicolor model, another commonly used lineage tracing model is the conditional gene editing model (Rosa26‐LSL‐Cas9 mouse model). The Rosa26‐LSL‐Cas9 mouse model was designed to achieve time‐specific and tissue‐specific gene editing by inserting LoxP‐Stop‐LoxP (LSL)‐Cas9 element at the Rosa26 site, combined with the conditional expression of Cre recombinase. The Rosa26 site is a commonly utilized safe harbor where inserted exogenous genes can be stably expressed in almost all cell types.^92^ This model contains Cas9 encoding gene with a LoxP‐Stop‐LoxP element inserted upstream to prevent Cas9 nuclease from being expressed when it is not needed.^93^ Only when Cre recombinase is expressed in a target cell, the Stop sequence is excised, and Cas9 is expressed, allowing cell‐specific gene editing.^94^ The Rosa26‐LSL‐Cas9 mouse model is widely used to study the function of specific genes in specific tissues and developmental stages. For example, effects of Cas9‐mediated gene editing in specific cells on cardiac development and function can be studied with heart‐specific expression of Cre recombinase.^95^ In addition, this model can be combined with other gene editing tools, such as CRISPR interference (CRISPRi) or CRISPR activation (CRISPRa), to achieve precise regulation of gene expression.^96^

The CARLIN (CRISPR array repair lineage tracing) mouse model is based on CRISPR‐Cas9‐mediated DNA repair by introducing unique barcodes at specific sites through NHEJ in the presence of Cas9. This model activates Cas9 expression at various time points by a Dox‐induced system to generate unique barcodes in the cellular genome that enables simultaneous query of lineage history and gene expression profiles at the single‐cell level.^5^ Using this model, researchers have induced expression of CRISPR‐Cas9 system at various stages of mouse embryonic development (e.g., E9.5) and generated up to 44 000 unique barcodes. Furthermore, scRNA‐seq analysis elucidated intrinsic bias in fetal liver hematopoietic stem cell (HSC) clones. Clonal bottlenecks in HSC response to injury were also identified. Thus, the application of CARLIN was able to study the clonal kinetics of blood replanted after chemotherapy and trace developmental pathways in various tissues,^97^ providing unprecedented insights into cellular heterogeneity and function in vivo.

The high reproducibility of developmental processes in multicellular organisms suggests that a set of systematic regulatory programs can control cell‐fate decisions. According to Waddington's theory of the epigenetic landscape, various cell types arise from unstable stem cell or progenitor cell states during fate determination and eventually fall into stable cell fates. Recent advancements in single‐cell genomics are altering our understanding of the Waddington landscape and abstracting a more comprehensive concept of “state manifold” to enhance understanding of lineage development. Fei et al. conducted single‐cell transcriptome analysis on 10 important tissues in seven important developmental stages of mice, spanning from early embryonic stage to adult maturity, covering nervous, respiratory, digestive, circulatory, urinary, reproductive, and other systems. More than 520 000 single‐cell transcriptome data were obtained, depicting the manifold of cell states of lineage differentiation during mouse development and revealing lineage‐common and lineage‐specific regulatory programs during cell‐type maturation. There were indications that lineage‐common regulatory programs are broadly active during the development of invertebrates and vertebrates. In particular, Xbp1 is an evolutionarily conserved regulator of cell‐fate determination across species. According to the analysis of common regulatory elements across lineages, Xbp1 transcriptional regulation is important for stabilization of the gene‐regulatory networks in a wide range of mouse cell types. This study offered genetic and molecular insights into cellular gene‐regulatory programs and will serve as a basis for further advancing the understanding of cell‐fate decisions.^98^

A fundamental interest in developmental neuroscience lies in the ability to map complete single‐cell lineages in the brain. Xie et al. developed a CRISPR editing‐based lineage‐specific tracing (CREST) method for clonal tracing in Cre mice. Using snapshotting CREST (snapCREST), they constructed a spatiotemporal lineage landscape of the developing mouse ventral midbrain (vMB) and identified six progenitor archetypes that could represent the principal clonal fates of individual vMB progenitors, as well as three distinct clonal lineages in the floor plate that specified glutamatergic, dopaminergic, or both types of neurons. They further developed pandaCREST (progenitor and derivative associating CREST) to associate transcriptomes of progenitor cells in vivo with their differentiation potentials. They identified multiple origins of dopaminergic neurons and demonstrated that a transcriptome‐defined progenitor type comprises heterogeneous progenitors, each with distinct clonal fates and molecular signatures. Therefore, the CREST method and strategies enabled comprehensive single‐cell lineage analysis, which could provide new insights into molecular programs underlying neural specification.^62^

### 4.5 Application of cell lineage tracing technology in porcine model

In the application of lineage tracing technology, porcine models, which are more similar to humans in body size and physiological characteristics, have had important roles in regenerative medicine, xenotransplantation, and the study of human diseases.^99^, ^100^ Using CRISPR‐Cas9 technology, researchers have been able to precisely insert or delete specific genes in the pig genome and thus study their roles in development and disease.^101^ Based on these advantages, several specific applications of porcine models in lineage tracing technology are described below.

Using CRISPR‐Cas9 technology, specific fluorescent protein‐encoding genes were introduced at the embryonic stage of pigs and expressed in specific cell types that could be tracked in cells and their offspring. For example, Whitworth et al. used CRISPR‐Cas9 technology to knock out the porcine CD163 gene generating a porcine model resistant to porcine reproductive and respiratory syndrome virus (PRRSV). Using fluorescent labeling, they were able to track distribution and immune response of edited cells in pigs.^102^ Similarly, Suzuki et al. used CRISPR‐Cas9 technology to edit multigenes related to fat metabolism in a porcine model and studied effects of these genes on fat deposition and metabolism.^103^ Using fluorescent labeling and barcode tracking, they analyzed effects of various edited genes on cell behavior.

In addition, researchers can establish pig models with pathogenic mutations in humans and use lineage tracing to track cell fate over the course of therapy.^104^ For example, in studies of muscular dystrophy, Moretti et al. used CRISPR‐Cas9 technology to introduce genetic mutations that can cause muscular dystrophy in a porcine model.^105^ Subsequently, they observed and tracked the repair and regeneration of muscle cells after treatment through gene therapy and evaluated the efficacy and safety of gene therapy. Furthermore, porcine models have been developed to mimic various human diseases, including neurodegenerative disorders,^106^ cancers,^107^ and gastrointestinal disorders.^108^ Scientists can better understand effects of gene therapy in large animal models, providing valuable preliminary data for clinical application.

### 4.6 Application of cell lineage tracing technology in nonhuman primate model

Nonhuman primates have similar characteristics in genetics, organ structure, physiological functions, pathological responses, and biochemical metabolism to humans. These primates are ideal animal models for biomedical research and drug development, with substantial research value. As a commonly used nonhuman primate model in biomedical research, *Macaca fascicularis* has been widely used in preclinical pharmacological and toxicological studies, including SARS‐CoV‐2. Therefore, systematic evaluation of differences in cell composition, organ heterogeneity, and spatiotemporal specificity of gene expression between nonhuman primate models such as *M. fascicularis* and humans will provide an important theoretical basis for in‐depth understanding of physiological functions, drug responses, disease prevention, and treatment related to human life activities.

Qu et al. mapped the major organs of nonhuman primates *M. fascicularis* (heart, liver, spleen, lung, kidney, stomach, colon, muscle, trachea, aorta, fat, tongue, breast, bladder, uterus, and testis) using scRNA‐seq and single‐cell chromatin openness sequencing (scATAC‐seq) to create the multiomics reference map of single‐cell transcriptome and interactome. Approximately 250 000 cells were clustered to obtain >40 distinct cell subsets, involving 17 major cell types.

Based on this map, researchers systematically compared cell differentiation and interactions of various organs. For example, ciliated cells are present in various organs of *M. fascicularis*, and characteristic genes of different ciliated cell subsets are enriched for various functions related to metabolic processes, signal transduction, and cellular responses to stimuli. By combining expression profiles and open chromatin data, researchers also analyzed and predicted key regulators controlling gene expression patterns in various cell types. For example, SPIB, POU2F2, SPI1, CEBPD, and IRF4 are key regulators in myeloid cells. The transcription factor (FEV) has potential to regulate hematopoietic stem cells and is widely involved in the regulation of gene expression in immune cells and epithelial cells. Researchers have constructed organ‐specific gene regulatory networks, which will provide more comprehensive information for understanding organ structure and function, exploring human disease mechanisms, and development of preclinical drugs.

In addition, through comparative analyses across species and integration analysis of single‐cell transcriptome data in matched organs, cell composition and gene expression were studied in humans, *M. fascicularis*, and mice. *M. fascicularis* and humans shared abundant types of immune and epithelial cells, with a high degree of similarity in cell composition, cell interactions, and correlations in gene expression patterns in matched organs. This study provided a theoretical basis for *M. fascicularis* as a model for immune‐related complex diseases.^109^

## 5 EVOLUTION OF CELL LINEAGE TRACING

Development and wide application of cell lineage tracing technology has greatly promoted advances in biomedical research. These technologies not only support detailed tracking and study of cell fates and evolution during development, regeneration, and disease but also provide powerful tools for gene therapy and regenerative medicine. Initially, imaging‐based methods for lineage tracing relied on direct observations through microscopy to track transparent invertebrate embryos, taking advantage of the small size, transparency, and determinate embryonic cleavage patterns of this species.^8^ More complex species (e.g., vertebrates) often contain a huge number of cells and indeterminate cell divisions, which are more difficult to observe directly. Lineage tracing thus expanded to a wide range of additional approaches, including the injection of dyes, cell transplantation, and in vivo genetic recombination methods.^110^ The most common imaging‐based method relies on transgenic fluorescent reporter genes to track cell states.^111^ However, the number of distinct cells that can be simultaneously queried using fluorescent reporters is intrinsically limited, and the above methods remain extremely challenging and may fail to capture all cells.

Recently, high‐throughput sequencing has enabled a new generation of lineage tracing approaches. These new methods use DNA sequence barcodes to encode information about cell identity. DNA sequence complexity scales exponentially with the length and multiplicity of the engineered barcodes, which is theoretically sufficient to allow a record of every single event in organism. The recorded information is read out using high‐throughput sequencing and can be readily combined with other sequencing‐based omics measurements. DNA barcoding technologies show considerable potential as lineage tracing tools. Given the rapid evolution of this field, it is helpful to appreciate the limitations that are likely to be resolved, as well as some methodological improvements that are already emerging.

Most CRISPR‐Cas9 barcoding methods rely on random indels introduced during the process of double‐strand break repair by NHEJ. CRISPR‐Cas9 activity has been shown to cause cell death in hiPSCs^112^ and cell lines^68^ and result in developmental delay in mouse embryos.^27^ The effect of potential off‐targets also remains generally unaddressed. Therefore, alternatives to CRISPR‐Cas9‐based methods may not face the same concern of excessive DNA damage and can avoid double‐strand breaks using genetic recombination,^12^, ^113^ CRISPR‐associated transposase systems (CAST and *Vibrio cholerae* Tn6677),^114^, ^115^ base‐editing enzymes,^63^, ^116^ prime editing,^117^ and other CRISPR‐system nucleases. Failure to detect edited barcode sequences can skew inferred lineage relationships, possibly due to low or noisy levels of barcode reporter expression or endogenous silencing of exogenous transgenes or lentivirus.^118^ Lineage tracing using DNA barcodes should assess the per‐cell barcode detection rate, which can be estimated in principle through control experiments in which lineage relationships can be independently verified.^119^ In addition, steps to improve experimental detection may be needed, for example, introducing strong RNA polymerase II promoters to drive transcription of mRNA‐based barcodes.

Importantly, lineage tracing methods should generate far more barcodes than the number of cells to be analyzed. Barcode diversities generated by CRISPR‐Cas9 have varied considerably. True barcode diversity, however, is likely to be overestimated, in part, because certain errors in double‐strand break repair reoccur frequently.^120^ In addition, generation of multiple DNA double‐strand breaks in close proximity leads to excision of intervening sequences, resulting in the loss of previously generated edits.^42^ Finally, the activity of DNA repair machinery may differ among organisms, tissues, and species. Approaches to expanding the diversity of Cas9‐editing barcodes include the expression of additional transgenic components at the time of barcoding with the use of terminal deoxynucleotide transferase (TdT) or increasing the number of barcoding events per cell, with improved integration rates to expand combinatorial diversity.^121^, ^122^ In CRISPR‐Cas9 systems, barcode diversity can be increased through parallel editing of several transgenic DNA target sites arranged in tandem or distributed throughout the genome.

Most recent methods use distributed barcode arrays, which greatly reduce the number of nucleotides that must be sequenced to recover the barcode identity and also provide the advantage of being far less susceptible to internal deletions and information loss.^27^, ^31^, ^121^, ^122^, ^123^ Additional CRISPR‐Cas nucleases, such as Cas12a, are substantially more specific than Cas9 and can result in more diverse editing outcomes. Multitarget Cas12a barcode designs are often employed to increase barcode capacity.^73^ In recombination systems, the number of barcode possibilities increases with the number of recombination sites. In the PolyLox system, nine loxP sites yield >1.8 million Cre recombination possibilities.^12^

A critical requirement for lineage tracing is the need to label small numbers of cells in a defined tissue of interest to ensure adequate sampling of their resulting progeny. In addition, interpretation of cell tracing experiments depends strongly on precisely controlling the time interval in which cells are labeled. Targeting labeling to specific tissues can be facilitated by expressing barcodes under the control of tissue‐specific promoters.^71^ In CRISPR‐Cas9 system, timing of barcoding can be fine‐tuned by adjusting availability of the three components. The direct way is to moderate the Cas9 activity by dox induction or heat shock induction, but a priori knowledge is required to select the best timing of barcoding.^5^ Confining the Cas9 activity in a specific phase of the cell cycle, such as the S/G2 phase, should slow down target exhaustion and eliminate postmitotic editing events that may confound the lineage inference.^124^ Although researchers have explored only DNA sequences as lineage barcodes, there is considerable potential for using other DNA features as lineage barcodes. For example, cytosine 5‐hydroxymethylation patterns were used to trace back sister cells in the mouse early embryo.^125^ Additionally, DNA methylation has been used to resolve clonal patterns in human fetal hematopoietic progenitor cells and in human adult leukocytes.^126^, ^127^, ^128^ It is highly likely that several other (epi)genetic marks could be used to link cells to their ancestors.

## 6 FUTURE APPLICATIONS: ORGAN TRANSPLANTATION

As an efficient gene editing tool, CRISPR‐Cas9 system has an important role in cell lineage tracing technology. This technology precisely tracks the fate of cells during development, regeneration, and pathology by introducing genetic markers such as fluorescent proteins or barcode sequences. Remarkable progress has been made in model animals that can reveal the basic mechanisms of biological processes. Organ transplantation is a vital medical technique for replacing or repairing organs that have lost function. However, major challenges faced by organ transplantation are shortages in donor organs and immune rejection.^129^ Combining CRISPR/Cas9 systems with lineage tracing technology has allowed more precise cell tracking and genetic modification in higher mammals such as pigs and sheep. By introducing specific genetic tags into the cells of the donor animals, researchers were able to track the behavior of these cells in real time in the recipient. This approach not only can assess the fitness and functional recovery of the transplanted organs but also elucidate possible problems during transplantation, leading to timely intervention and adjustment to improve the success rate of transplantation.^130^

**FIGURE 3:**
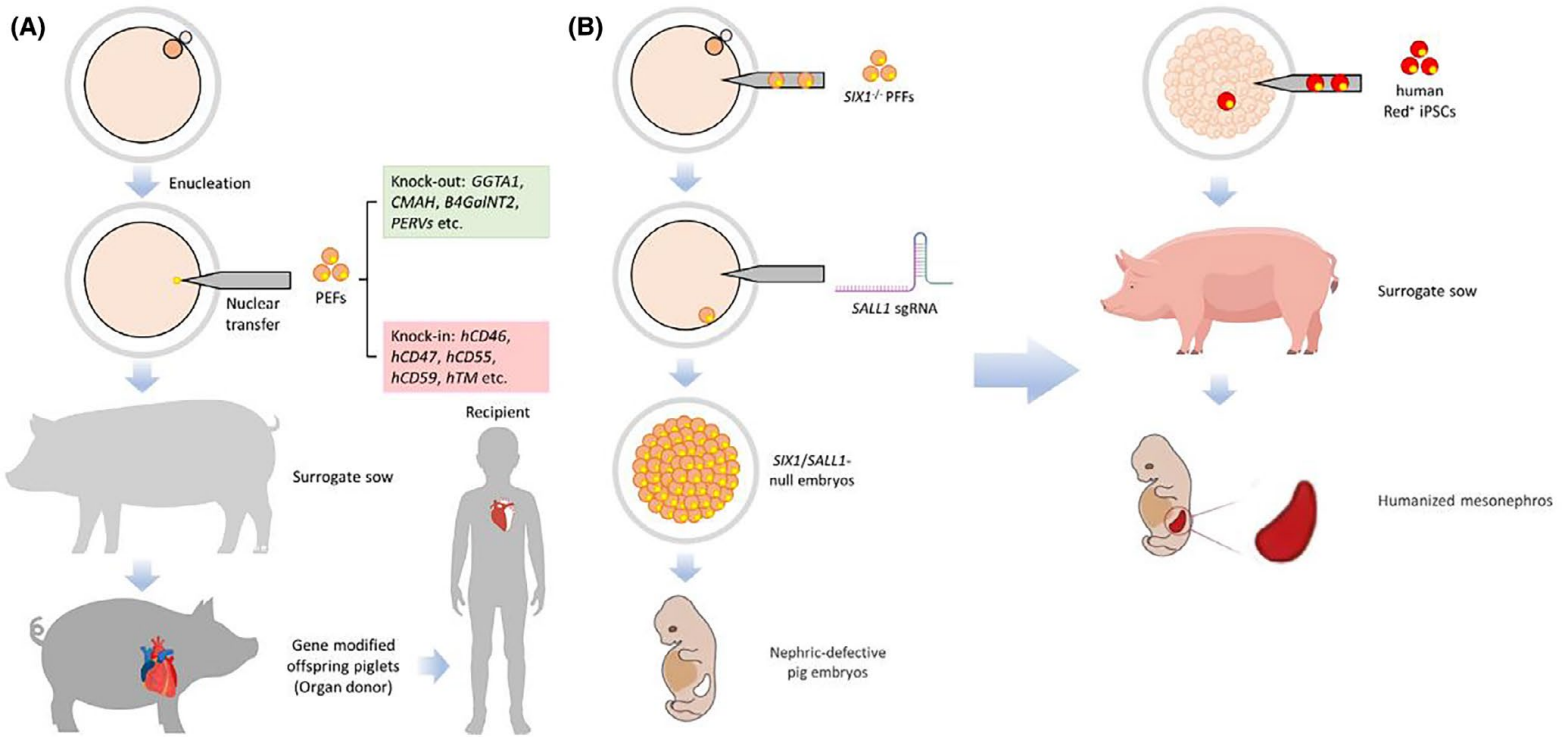
The pig models for xenotransplantation and xenogeneic organ reconstruction. The preparation process of pig model for xenotransplantation is shown in (A). The gene modified pigs were generated by deleting genes involved in immune rejection (e.g., alpha‐Gal) and endogenous viruses (PERVs) and introducing genes for human complement regulatory proteins (e.g., CD46, CD55, and CD59) to reduce immune rejection and improve physiological compatibility. The gene‐modified pigs derived from somatic cell nuclear transfer reprograming technology can be used as an ideal source of human organ donors. The strategy of pig model for xenogeneic organ reconstruction is shown in (B). By knocking out SIX1 gene that regulates ureteric bud branching and SALL1 gene that regulates metanephric interstitial development, a nephric‐defective pig model with a more adequate organ niche, as well as mesonephros development defect and metanephros complete absence, was constructed. Human pluripotent stem cells developed in nephric‐deficient embryos and were able to generate a human mesonephros in a nephric‐deficient porcine model, which was successfully embedded in the porcine mesonephros.

Compared to traditional model animals such as mice and rats, a pig model has unique advantages as a model animal for clinical trials. Large animals, such as pigs, are used as an ideal source of organ donors because they have a high similarity to humans in organ size, physiological and biochemical characteristics.^131^ At the beginning of 2022, successful transplantation of a pig heart to human by American scientists caused a huge sensation; xenotransplantation has moved from laboratory to clinic! Although the transplant recipient only survived for 60 days, it was of epoch‐making significance. The application of pigs as xenotransplantation organ donors requires genetic modification of some immune rejection genes, and 10 genetically modified pigs were used in this project. Genetically modified pigs can reduce immune rejection and improve physiological compatibility by deleting genes involved in immune rejection (e.g., alpha‐Gal) and introducing genes for human complement regulatory proteins (e.g., CD46, CD55, and CD59) to provide a new solution for xenotransplantation.^132^ In addition, more modifications for immunogenes in pigs may be more beneficial for survival of xenotransplantation recipients. Researchers are exploring the mechanism of the innate immune receptor toll‐like receptors in the process of immune rejection.^133^, ^134^ The use of genetically modified pigs to overcome immune rejection, endogenous viruses, and other challenges should prolong survival of xenotransplantation recipients (see Figure 3A). However, there are still many difficulties.^135^ There are several key approaches beyond genetic modification that may address immune rejection and other associated challenges to improve the success rate of xenotransplantation potentially and broader clinical application in the future.^136^

First of all, PERVs, which are a concern in pig‐to‐human transplants, must be inactivated to prevent cross‐species infection.^137^ Next, more effective and targeted immunosuppressive drugs, which can prevent rejection and techniques to induce immune tolerance (e.g., mixed chimerism, where donor and recipient hematopoietic cells coexist), need to be developed.^100^ Using nanoparticles to deliver immunosuppressive drugs directly to the site of the transplant may minimize systemic side effects.^138^ Finally, tailoring personalized medicine based on the individual's immune profile and response to the transplant is also very important. It should be noted that treatments need to be adjusted promptly based on better biomarkers for early detection of rejection and real‐time monitoring of the immune response. In addition, biological engineering methods, including decellularization of donor organs and recellularization with the recipient's cells^139^ and bioengineered organs using three‐dimensional (3D) bioprinting and the recipient's own cells, can reduce immunogenicity.^140^ Cellular therapy, including stem cell^141^ and regulatory T cells (Tregs)^142^ modulating the immune response, can reduce the risk of immune rejection and promote tolerance to the transplanted organ.

In 2010, Kobayashi et al. injected rat iPSCs into Pdx1 knockout mouse embryos with pancreatic dysplasia, and the descendants developed pancreases derived from rat iPSCs. The reconstructed organ had normal function and was fully capable of compensating for the function of the deficient mouse pancreas. This study confirmed the possibility of xenogeneic organ reconstruction and described the first application of blastocyst complementation method in xenogeneic organ reconstruction.^143^ The core of this approach is generation of embryos with defective organ development by gene knockout and replacement of the lacking organ by injection of exogenous pluripotent stem cells.

In 2017, Wu et al. demonstrated that human iPSCs could form chimerism with early porcine embryos, and the chimerism rate of human stem cells in porcine embryos after implantation was 1 in 100000.^144^ Despite the low chimerism rate, chimerism of human‐derived stem cells in postimplantation porcine embryos shows the feasibility of xenogeneic organ reconstruction in more complex species. In 2023, Wang et al. combined a new gene editing tool with nuclear transfer technology and embryo micromanipulation technology to further advance this field. By knocking out SIX1 gene that regulates ureteric bud branching and SALL1 gene that regulates metanephric interstitial development, they constructed a nephric‐defective pig model with a more adequate organ niche, as well as mesonephros development defect and metanephros complete absence, which created a suitable ecological environment for human kidney development in pigs. Human pluripotent stem cells developed in nephric‐deficient embryos for 28 days and were able to generate a human mesonephros in a nephric‐deficient porcine model, which was successfully embedded in the porcine mesonephros and resulted in the formation of mesonephric tubules, forming a structurally intact defective kidney^145^ (see Figure 3B).

Based on current results, future research will be further developed by applying lineage tracing technology to track migration and differentiation of key cells during embryonic development, and then using single‐cell sequencing technology to construct a detailed developmental lineage tree, thereby identifying core genes that play key roles in organ formation. Furthermore, research will continue to optimize the organ deletion model, use more precise gene editing technology (such as CRISPR‐Cas9) to generate “empty” embryos with higher chimeric efficiency, and improve the ability of exogenous pluripotent stem cells to generate functional organs in recipients by regulating expression of developmental genes.

The application of blastocyst complementation method will also be further extended, especially in complex large animal models, such as pigs and monkeys, to explore reconstruction of complex organs (e.g., heart and kidney). To reduce immune rejection after transplantation, regulation of host immunogenes will also be further improved. Ultimately, the incremental development of these technologies will promote clinical translation, perform preclinical validation of xenotransplantation, and ensure its feasibility in terms of function, safety, and long‐term survival, thereby providing an effective and innovative solution to the global organ shortage.

The current cell lineage tracing experiments focus on embryogenesis and hematopoiesis; however, future interrogations should witness its broad application in animal development, regeneration, tumorigenesis, and stem cell dynamics. Regardless, obtaining an accurate and systematic lineage tree of a species remains a challenging task, and new designs are necessary for further improvements.^146^, ^147^, ^148^ Recently, development of spatial transcriptomics has shed light on the understanding of the spatial organization of cell types and their lineage relationships.^149^

In the last few years, technologies utilizing organoids have developed rapidly. As human stem‐cell‐derived organoids could mimic the organ or tissue development of humans, cell lineage tracing on human organoids may provide a basis for studying lineages of human cell types. Cell lineage tracing on human organoids displays the following advantages. First, specific organoids might describe the lineage transition of human cell types more accurately and controllably than mouse in vivo equivalents, especially for complex organs such as the human brain.^150^ Second, manual modulations of culture conditions enable studying phases of organ development step by step. For example, current breakthroughs allow for studying cardiogenesis at multiple overlapping stages such as gastruloids, foregut‐heart organoids, or heart organoids.^151^, ^152^, ^153^, ^154^ Third, because organoids are cultured in a dish, it's convenient to combine cutting‐edge imaging techniques to track cell behaviors. In conclusion, complementary studies of embryonic development and organoid development could provide a clearer picture of cell‐fate decisions in the future.

## 7 SUMMARY

Although research using CRISPR‐Cas9 system for lineage tracing in higher mammals is still in its infancy, it is expected to achieve breakthroughs in improving tracking accuracy, dynamic recording, and multiple labeling, with great potential in organ transplantation and disease research. Through combinations of gene modification and advanced gene editing technology, more accurate and efficient lineage tracing in large animals can be achieved, promoting progress in xenotransplantation and other fields. Future research and technological improvements are expected to overcome the current challenges and achieve more ideal xenotransplantation, which will also bring new prospects for regenerative medicine and personalized medicine.

## AUTHOR CONTRIBUTIONS

**Jingchao Cao:** Data curation; writing – original draft. **Zihang Guo:** Data curation; writing – original draft. **Xueling Xu:** Writing – review and editing. **Pan Li:** Writing – review and editing. **Yi Fang:** Writing – review and editing. **Shoulong Deng:** Supervision; writing – review and editing.

## FUNDING INFORMATION

This work was supported by Institute of Laboratory Animal Sciences, Chinese Academy of Medical Sciences and Comparative Medicine Center, Peking Union Medical College, Collaborative Innovation Program of the Chinese Academy of Sciences (22SH19), and Non‐profit Central Research Institute Fund of Chinese Academy of Medical Sciences (2023‐PT180‐01).

## CONFLICT OF INTEREST STATEMENT

The authors declare no conflicts of interest.

## ETHICS STATEMENT

This review did not involve human or animal subjects, therefore ethics review was not applicable.
